# The birth of *Emerging Themes in Epidemiology*: a tale of Valerie, causality and epidemiology

**DOI:** 10.1186/1742-7622-1-1

**Published:** 2004-10-06

**Authors:** Clarence C Tam

**Affiliations:** 1 on behalf of the editorial board of Emerging Themes in Epidemiology; 2 Deputy Editor, Emerging Themes in Epidemiology; 3 Infectious Disease Epidemiology Unit, Department of Infectious & Tropical Diseases London School of Hygiene & Tropical Medicine, London, United Kingdom

## Abstract

*Emerging Themes in Epidemiology (ETE) *is a new, online, Open Access peer-reviewed journal. The Journal is unique in that it was conceived and is managed by research degree students in epidemiology and related public health fields. The Journal's management is overseen by its Editor-in-Chief and Associate Faculty Editors, all of whom are senior members of faculty. *ETE *aims to encourage debate and discussion on the theoretical, methodological and practical aspects of epidemiologic research and practice. In addition, *ETE *is dedicated to the promotion of Open Access publication and the training of research students in the scientific publishing process. This editorial, to coincide with the launch of *ETE*, sets out the Journal's philosophy and aims. Epidemiology is a rich and innovative science that has much to gain from broader discussion of the causal frameworks that underpin it. *ETE *aims to be a major forum for such discussion.

## 

"*Causality. There is no escape from it, we are forever slaves to it. Our only hope, our only peace is to understand it, to understand the 'why'*[1]."

- The Merovingian

As far as we know, the Wachowski brothers' Merovingian was not an epidemiologist, but his sentiments should resonate well with those in the field. Probably more than those in any other profession, epidemiologists are slaves to causality. Our professional lives are dedicated to the pursuit of pumphandles, spiderwebs and causal pies [2,3]. Adding to these colourful metaphors are ones proposing frameworks for epidemiologic research that involve Chinese boxes, computer-generated fractals and prison breaks [2,4,5]. But what is the student of epidemiology to make of all these curious abstractions? Lost in a sea of metaphors, they might very likely throw their arms up in the air and, in thorough confusion, decide to take a long and much-needed coffee break [6-8].

The epidemiologic literature on causality certainly makes for stimulating reading, but it would be interesting to know how many of us have causal pies and fractals on our minds as we reach for that red folder labelled "Logistic regression 101". Discursive articles on the usefulness of such metaphors are widely regarded as philosophical flights of fancy that we might eventually get around to reading after clearing that backlog of papers waiting to be written in the next two months. Yet what are our alternatives? The newly-qualified epidemiologist leaves their degree with a solid grasp of error, bias, confounding and statistical methodology, but with perhaps a single lecture on Koch-Henlé postulates and Bradford-Hill 'criteria' as the extent of their training into causal thinking. It is interesting to note that Last's *Dictionary of Epidemiology *does not include the term 'cause', opting instead to give a definition of 'causality' that involves a brief discussion of necessity and sufficiency [9]. Any reasoned assessment will quickly lead to the conclusion that guidelines for determining causal pathways as commonly taught in epidemiology courses are woefully inadequate, regardless of whether one decides to take an inductionist, refutationist, or hypothetico-deductivist view [10,11], or admits to not having a clue what any of these terms mean. The challenge for the modern epidemiologist is to put those newly-learned methods to use from within a causal limbo, with Robert and Austin as their guides and John Snow as spiritual counsellor.

## The anatomy of a cause

James Wong's 2000 teen horror movie *Final Destination *[12] is unlikely to go down in history as a cinematic classic, but it is memorable for its clever, if rather gruesome, depictions of causal processes leading invariably to the death of a number of its unfortunate characters. The movie's motto is that "you can't cheat death's plan". Having narrowly avoided a fatal plane accident thanks to the protagonist's chilling premonition, a French teacher and five of her students are destined to fall victims to the Grim Reaper one by one- in the order they would have died had they been on the ill-fated plane with the rest of their class- unless they can find a way to break the cycle and cheat death. In the most elaborate death scene, Valerie, the French teacher, alone in her house and still visibly shaken by the loss of her colleague and students, becomes unnerved by noises outside. With John Denver's *Rocky Mountain High *ironically playing in the background, she tries to calm herself by making some tea. Moving to the kitchen sink, she fills the kettle with water, spilling some down the side. She wipes the kettle, turns towards the gas stove and tosses the towel carelessly behind her, which catches onto a knife block. With the kettle whistling, she picks up a school coffee mug, drops two tea bags inside and fills it with boiling water. Picking up the mug, she suddenly recognizes the school logo and, in shock, reflexively throws the mug's contents into the sink. Opting now for something stronger to calm her nerves, she takes some ice cubes from the freezer, drops them into the still-warm mug and re-fills it, this time with vodka. Oblivious to the crack that has appeared in the mug, she walks towards the living room, leaving a trail of vodka behind. As she stands by her computer monitor, vodka drips into the circuitry. An electrical surge creates a spark that ignites the alcohol, causing the monitor to explode and sending out flying shards of glass that slash her throat. Shocked and bleeding, she stumbles towards the kitchen sink, chased by a trail of burning alcohol. Reaching the stove, the trail of flames ignites the gas burners, lighting up her clothes and hair. Falling to the ground and still bleeding, she rolls around violently trying to put out the flames. In an act of desperation, she reaches up and grabs the dangling towel, tilting the knife block and sending a half dozen blades cascading into her stomach while flames catch the curtains and set the house on fire.

Suppose you were the investigator arriving on the scene. There is a half burnt-down house, a blown-up computer, a broken mug and a corpse with third-degree burns, stab wounds and a cut throat, but no signs of struggle. What would you determine was the cause of this tragedy? The severe burns, the protruding knives and the neck wound would be pretty obvious choices. But perhaps it is more complicated than that. Perhaps there were extenuating circumstances without which this tragic outcome might not have occurred. The exploding computer maybe, or what about the vodka that burned leaving no trace, or the cracked mug? No, maybe it was the towel, complicitly catching onto the knife block. Or maybe we should blame the teacher's drinking habit.

The point of this rather unsavoury story is that without the benefit of such extrinsic observation, detailed reconstructions of causal processes are unattainable. A reasoned observer might conclude that all of the above factors were in some way responsible. They all contributed to the process in their own small way, and had any one of them not been involved things may not have turned out the way they did. They were all what one might call 'component causes'. But is this enough to convince us of what the real cause was? Clearly not.

Suppose now that you were an audience member and were somehow able to communicate directly with the characters in the movie. You might wish to warn Valerie of her impending ill fate. At which point in the whole sequence would you alert her so that her death could be prevented? Clearly you would not deny her a mug of tea and you would most likely have no way of knowing that the towel would land on the kitchen block with dire consequences just a few moments later. You might, of course, realize this at the last moment and warn her against reaching up and grabbing the towel, but she might still have died from her neck wound. You might have shouted for her to grab a fire extinguisher and put out the trail of flames, but it is unlikely she would have listened to you as she tried hopelessly to stop the bleeding from her neck. You might, with better foresight, have pointed out to her that vodka was dripping from her mug. Or perhaps with hindsight, you might have recognized that the best thing would have been to provide some moral support and consolation in her time of grief, with which the whole sorry incident might have been avoided altogether.

One thing is now clear. There are steps within causal processes on which we can act to try and alter their course- the trail of flames might have been extinguished, and the consequences of the dripping vodka might have been avoided. There are other steps on which we can have no influence, eg. the tossed towel landing on the knife block. Another important thing is also apparent: causal processes have hierarchies. Depending on what happens at one stage, a number of alternative events may result at the next. Had Valerie been able to stop the trail of flames, she might have reached the kitchen sink, realized she could not stop the bleeding and called an ambulance. Or she might have run out of the house shouting for help and her neighbour, trained in first aid, might have saved her life. Saving victims of horror movies, however, is not an easy job. Knowing when best to step in is not necessarily that simple, as we have seen with Valerie. In some cases we may be given a number of opportunities and intervening at any of them might lead to a positive outcome. But in other cases, once certain factors are in place there will be an air of inevitability in everything that follows, and all our attempts to intervene thereafter may prove to be little more than an exercise in futility.

Perhaps it is now time to admit that I have extended this fanciful analogy far beyond what is appropriate. I make no apology if in so doing I have in some way managed to convey the idea that epidemiology thrives on causal processes, on elucidating their complexities and identifying the most effective points for intervention no matter at what level of their elaborate hierarchies. If this is a worthwhile venture, then the discussion of how we conceptualize and study these causal processes surely is so too.

*Emerging Themes in Epidemiology (ETE) *was born out of this ideal- that contrary to common belief, epidemiology is not merely a collection of standardized tools to be applied at will to any health-related situation, but that it is a rich and innovative science that aims to describe reality in all its complexity, spanning the molecular to the global, with the ultimate goal of improving the health of individuals and populations. And that in order for this to be achieved, we need to improve our understanding of how factors, at any level of biological or social organization, eventually lead to ill health.

*ETE *is founded on three core principles:

• That epidemiology and epidemiologists have much to gain from a broader and more fundamental discussion of the concepts and theoretical frameworks that underpin the practice of epidemiologic research

• That Open Access publication has a crucial role to play in reducing the current inequities in access to scientific information, which should be a universal and freely-available resource for the benefit of the whole of society

• That students of epidemiology and related fields can make substantial contributions to the introduction of new concepts and ideas into mainstream epidemiologic research, not only through writing, but also through having a direct influence over what is published

In keeping with this philosophy, we recognize that epidemiology has much to gain from developments in other fields and we welcome contributions from all public health professionals. We will consider articles that comment critically on current epidemiologic theory and practice, either generally or within a specific specialty, including articles from other fields that have implications for the conduct of epidemiologic research. *ETE *will not generally publish research reports, although exceptions may be made in cases where the results can be placed within a broader public health context to present a new concept or theoretical framework.

By making all of this material freely available online under the auspices of the Open Access publisher, BioMed Central, we aim to make *Emerging Themes in Epidemiology *a global forum for the discussion of new developments in epidemiologic thinking and practice that will benefit the global public health community. In doing so, we recognize that there is much that the scientific community can do to support Open Access publication. The health consequences of inequitable access to scientific information remain largely ignored, yet for years we have adhered to a system of publication that is restrictive and largely subsidized by institutions and libraries at great expense. Open Access is an important step towards making the publication process, and its associated costs, more transparent. We thus call on individuals, institutions, funding bodies and governments to engage in the Open Access movement by promoting and supporting Open Access publication. This will involve a major shift not just in publishing costs, but also in thinking. Our current measures for assessing the impact of academic research are inherently intertwined with our inequitable publication tradition. Developing new ways of assessing the quality of scientific research that are independent of journals' perceived 'impact' are imperative for the wider recognition of Open Access.

In promoting the role of students in the publication process, we intend for *ETE *to be a training ground for postgraduate students, providing them with an opportunity to be involved at every stage of the publication process, including commissioning, reviewing and writing articles. Our editorial board is formed principally of research students, who operate the Journal with support from an international group of associate faculty. Our collaborations extend to a growing number of students from diverse institutions serving as article referees. We welcome suggestions for extending these collaborations in the future.

The third millennium has brought with it exciting challenges for epidemiologists. An explosion in the availability of genetic and molecular information, the development of bioinformatics, the increasing application of sophisticated statistical analyses to model complex systems and the gradual incorporation of sociological approaches to understanding health inequalities have arrived together with sobering statistics on the state of global public health. Knowing when and how to apply these and other developments at a time of rapid political, social and biological change while maintaining clear sight of our public health goals will be the major challenge for current and future generations. It is our hope that *Emerging Themes in Epidemiology *will be a tool for epidemiologists confronting that challenge.
